# A Reliable and Robust Method of Measuring Male Pelvic Floor Muscle Volume Using Three-dimensional Computed Tomography

**DOI:** 10.14789/ejmj.JMJ24-0027-OA

**Published:** 2024-12-31

**Authors:** FUMITAKA SHIMIZU, ABULAITI ABUDUREZAKE, MYRIAM DIABANGOUAYA, YASUNARI TANAKA, TAKURO KOBAYASHI, HISAMITSU IDE, YOSHIFUMI TAMURA, SHIGEO HORIE

**Affiliations:** 1 Department of Urology, Juntendo University Graduate School of Medicine, Tokyo, Japan; 1 Department of Urology, Juntendo University Graduate School of Medicine, Tokyo, Japan; 2 Sportology Center, Juntendo University Graduate School of Medicine, Tokyo, Japan; 2 Sportology Center, Juntendo University Graduate School of Medicine, Tokyo, Japan; 3 Department of Advanced Informatics for Genetic Diseases, Juntendo University Graduate School of Medicine, Tokyo, Japan; 3 Department of Advanced Informatics for Genetic Diseases, Juntendo University Graduate School of Medicine, Tokyo, Japan; 4 Department of Digital Therapeutics, Juntendo University Graduate School of Medicine, Tokyo, Japan; 4 Department of Digital Therapeutics, Juntendo University Graduate School of Medicine, Tokyo, Japan; 5 Department of Sports Medicine and Sportology, Juntendo University Graduate School of Medicine, Tokyo, Japan; 5 Department of Sports Medicine and Sportology, Juntendo University Graduate School of Medicine, Tokyo, Japan; 6 Department of Metabolism and Endocrinology, Juntendo University Graduate School of Medicine, Tokyo, Japan; 6 Department of Metabolism and Endocrinology, Juntendo University Graduate School of Medicine, Tokyo, Japan

**Keywords:** pelvic floor muscle, three-dimensional computed tomography, workstation

## Abstract

**Objectives:**

The pelvic floor muscle (PFM) plays a major role in sexual and urinary functions. No objective method exists to measure the PFM in male. This study evaluated the reliability of male PFM volume using three-dimensional computed tomography (3D-CT).

**Methods:**

PFMs of five patients aged 43-83 years were selectively extracted from thin-slice CT using a workstation to reconstruct stereoscopic images and measure PFM volume. Two raters measured the PFM volume three times in all patients to confirm the reliability of PFM volume measurement. Intra- and inter-rater correlation coefficients, i.e., intraclass correlation coefficient, were determined. The CT attenuation of PFMs was also evaluated.

**Results:**

Raters 1 and 2 reported an average PFM volume of 46.4 ± 11.5 cm^3^ and 46.1 ± 12.5 cm^3^, respectively. The intra- and inter-rater correlation coefficients were 0.99 and 0.98, respectively. The average CT value of PFMs had a minimum of 13.7 Hounsfield Unit (HU) in the eldest male and a maximum of 38.9 HU in the youngest male.

**Conclusions:**

Male PFMs could be selectively extracted using a workstation to reconstruct a stereoscopic image. The PFM volume measurement is feasible and highly reproducible. To our knowledge, this is the first study that standardizes the method for measuring the male PFM volume using 3D-CT and examines its reliability.

## Introduction

The pelvic floor muscles (PFMs), together with the diaphragm, multifidus, and transversus abdominis, constitute an inner unit of the deep trunk muscles, involved in regulating body stability and abdominal pressure. The PFMs are comprised mainly of the levator ani muscles with somatic innervation from the lumbosacral plexus^1)^. PFMs play a major role in urinary and sexual functions. Pelvic floor muscle training (PFMT) has been reported to improve sexual function and urinary incontinence in women^2, 3)^. The biofeedback-assisted PFMT has been reportedly useful in confirming whether PFMT is performed accurately^4)^. Conversely, a meta-analysis involving women with overactive bladder showed the usefulness of pelvic electrical stimulation, but not the superiority of biofeedback-assisted PFMT^5)^. Some investigators reported the lack of well-designed studies to assess theoretical mechanisms and recommended assessing PFM volume and intensity pre- and post-intervention^3, 6)^.

In men, PFM exercises have also been reportedly useful in sexual function and urinary incontinence after robot-assisted radical prostatectomy (RARP)^7, 8)^. Conversely, some investigators reported these symptoms have been not improved with PFMT^9)^. As with studies of female PFMs, several studies did not use objective measures to evaluate male PFM function, such as outcome measures being evaluated only with quality of life questionnaires.

The PFMs are involved in sexual and urinary functions; however, objective measures of their evaluation have not yet been established. Additionally, as far as we searched, there are no studies that objectively evaluate male pelvic organ prolapse (POP). We have a hypothesis that POP exists not only in women but also in men. Herein, evaluation of the PFM volume by 3D-CT using a workstation and the other male pelvic floor parameter were planned. The primary aim of this study was to evaluate the reliability of PFM volume measurement in men.

## Material and Methods

This retrospective study adhered to the guidelines established by the Declaration of Helsinki and was approved by the Institutional Review Board of Juntendo University School of Medicine (approval no. E23-0404-H01). For retrospective medical record surveys that handle only existing samples, instead of omitting informed consent, information on the study implementation, including the purpose, is posted by the Department of Urology on the Juntendo University website. This work was supported by JSPS KAKENHI Grant Number JP23K10436.

This study standardized the method of measuring PFM volume using 3D-CT and verified its reliability.

### Patients

The urology outpatient population was retrospectively searched for men with 0.5-0.8-mm slices of abdominal-to-pelvic conventional CT (320-row multidetector Aquilion ONE ViSION Edition, Canon Medical Systems, Ohtawara, Japan) obtained between January 2021 and December 2023. The required sample size was five with a power of 0.8, a two-sided significance level of 0.05, an assumed intraclass correlation coefficient (ICC) of 0.8, and three measurements. Five adult male patients were selected, excluding those with a history of pelvic surgery among outpatients. All CT scans were performed in the supine position.

### 3D-CT assessment of the PFM

All CT data were imported into a workstation software application, Attractive Medical Image Processor (PixSpace Co. Ltd., Kitakyushu, Japan)^10)^. The PFM was selected and extracted to avoid overlapping surrounding organs in each slice. The contour trace function was used to reconstruct the PFM. The 3D volumetry function was used to measure volume. The following rules were used to standardize the selective extraction of the PFM.

**Figure 1:**
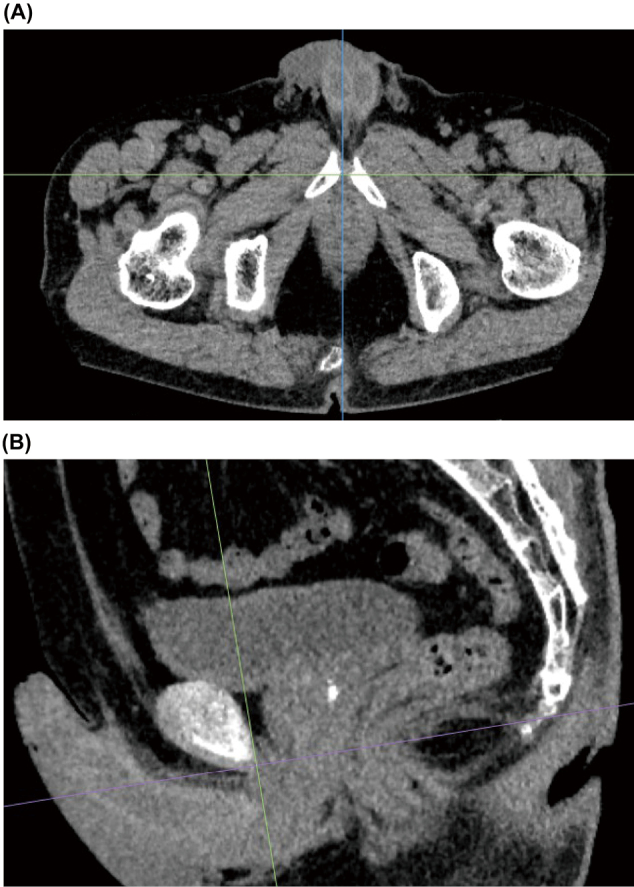
Setting up the oblique axes for measuring PFM volume at a workstation. (A) The midline was aligned in an axial image. (B) In the sagittal image, the line was drawn from the inferior border of the pubic symphysis to the posterior wall of the rectum at the anorectal junction (ARJ).

**Figure 2:**
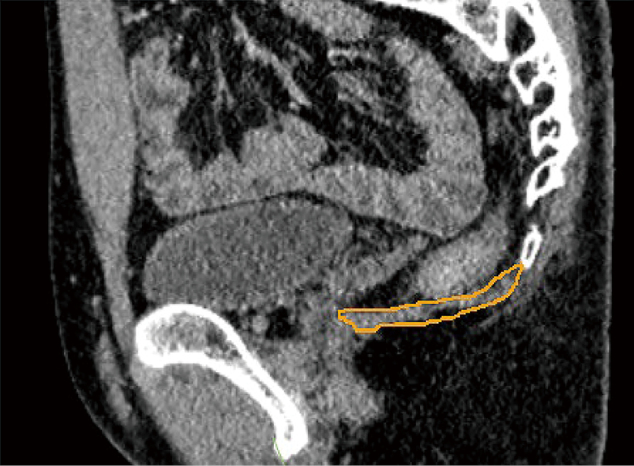
Selection of the deep PFM in a sagittal image

**Figure 3:**
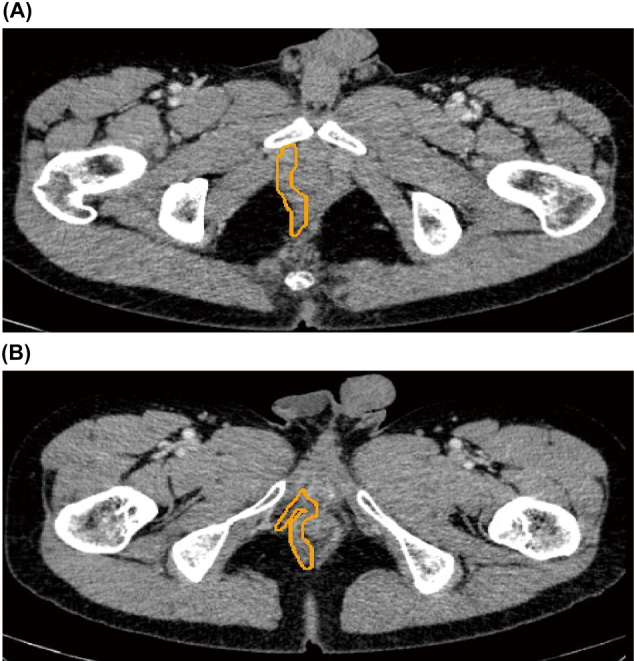
Selection of PFM in oblique axial images. (A) The deep PFM around the prostate and rectum was selected. (B) The superficial PFM was selected.

Oblique axial images were set as the basic axis for measuring PFM volume (Figure 1). The plane, which was considered parallel to the puborectalis muscle, was reconstructed to pass through the line from the inferior border of the pubic symphysis to the posterior wall of the rectum at the anorectal junction (ARJ), as described in a previous study^11)^. The deep PFM was selected in a sagittal image (Figure 2). Oblique axial images were used to select superficial and deep PFMs around the prostate and rectum (Figure 3). The zoom function was used more carefully as guide in selecting locations in which PFM was attached to the prostate and rectum. The volume rendering display of the used software was adjusted to prevent the visualization of fat surrounding the muscles. By selectively extracting the set of muscle voxels, the fat component was excluded from the volume.

**Figure 4:**
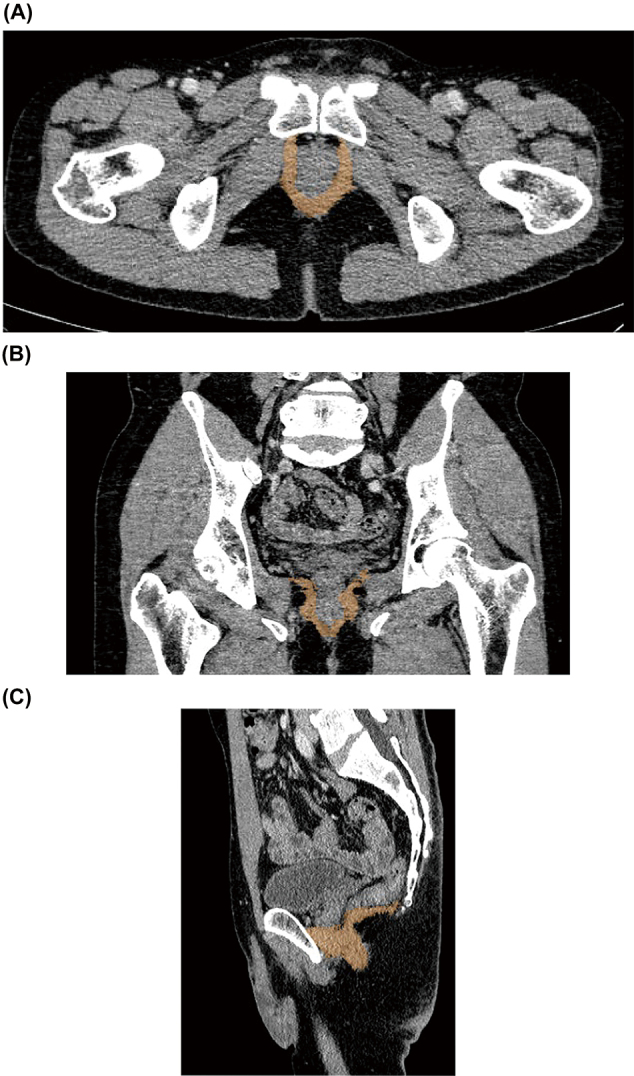
Images in which fat is removed and PFM is extracted. (A) Oblique axial images are shown. (B) The coronal section image is shown. (C) The sagittal section image is shown.

The muscle threshold range of the CT value is -29 to 150 Hounsfield Unit (HU)^12)^. Puborectalis, pubococcygeus, iliococcygeus, coccygeus, bulbospongiosus, transverse perineal muscle, and anal sphincter were extracted by these manipulations. The ischiocavernosus and urethral sphincter were not selected. Figure 4 shows the PFM images of oblique axial, coronal, and sagittal sections extracted after removing fat. To confirm the reliability of PFM volume measurements on CT, measurements were performed three times by two raters in all patients. The raters were board-certified physicians with 9 and 23 years of experience in urology.

### Other pelvic floor parameters

The average CT value of the extracted PFM was calculated. The area of the levator hiatus (LH) was measured in an oblique axial view of the plane connecting the inferior border of the pubic symphysis and ARJ. The vertical length of LH was defined as the H line, and the width of the LH was defined as WLH. The pubococcygeal line (PCL), widely used as a reference line, was drawn from the inferior border of the pubic symphysis to the last coccygeal joint. The distance from the PCL to the bladder neck, known as the B line, and to ARJ, known as the M line, were measured. The PCL angle from the horizontal was defined as the PCL angle.

### Statistical analysis

Intra- and inter-rater coefficients, i.e., the ICC, were determined. The one-way random effects model was applied to the intra-rater coefficient. The two-way random effects model was used for the inter-rater coefficient. ICC was interpreted as poor (0-0.49), moderate (0.50-0.74), good (0.75-0.89), and excellent (≥0.9) following Koo and Li's guidelines^13)^. For other pelvic floor parameters, the average, maximum, and minimum values were calculated. Statistical software, JMP17.0.0 (SAS Institute Inc., Cary, NC, USA), was used for statistical analyses.

## Results

The average age of five men was 71.2 years, with the youngest being 43 and the oldest 83. Table 1 shows detailed measurement results and average values using the workstation software PixSpace. The maximum and minimum PFM volumes were 66.7 cm^3^ and 30.9 cm^3^, respectively. Patient 1, the youngest male, had the largest PFM volume. Raters 1 and 2 reported an average PFM volume of 46.4 ± 11.5 cm^3^ and 46.1 ± 12.5 cm^3^ in five patients, respectively. The intra- and inter-rater correlation coefficients were 0.99 and 0.98, respectively. Table 2 shows the other pelvic floor parameters. The average CT value of the PFM had a minimum of 13.7 HU in the eldest male and a maximum of 38.9 HU in the youngest male. The minimum and maximum values of the area of the LH were 9.4 cm^2^ and 16.8 cm^2^, respectively. Patient 1, the youngest male, had the highest average CT values and PCL angle, and the lowest the area of LH, WLH, H line, and M line. Only the youngest male had a negative M line.

**Table 1:** PFM volume measurement results for two raters using the workstation software PixSpace

Males (n = 5)								
	Rater 1				Rater 2			
Subject	First(cm^3^)	Second(cm^3^)	Third(cm^3^)	Mean(cm^3^)	First(cm^3^)	Second(cm^3^)	Third(cm^3^)	Mean(cm^3^)
1	66.1	64.8	65.1	65.3	67.0	68.6	68.3	68.0
2	46.0	46.9	45.8	46.2	43.5	44.5	41.1	43.0
3	43.3	42.4	42.8	42.8	41.3	44.0	40.9	42.1
4	46.2	48.0	46.6	46.9	45.4	47.3	46.1	46.3
5	31.8	31.2	29.6	30.9	29.4	32.1	31.3	30.9
Mean (SD)(cm^3^)	46.7	46.7	46.0	46.4 (11.5)	45.3	47.3	45.5	46.1 (12.5)

**Table 2:** Other parameters related to the PFM

Subject	Average CT value of the PFM (HU)	The area of the LH (cm^2^)	WLH(cm)	H line(cm)	B line (mm)	M line (mm)	PCL angle(°)
1	38.9	9.4	2.6	4.7	-25.1	-5.3	27.7
2	20.4	16.8	4.2	5.5	-13.3	19.7	11.3
3	28.6	14.2	2.9	6.7	-26.7	8.8	25.2
4	13.7	15.8	3.5	6.0	-23.1	17.6	3.9
5	19.7	10.5	2.9	5.4	-33.5	4.0	13.8
Average	24.3	13.3	3.2	5.6	-24.3	12.5	16.4

Abbreviations: LH, levator hiatus; WLH, width of the LH; PCL, pubococcygeal line

## Discussion

The pelvic floor is composed of muscles, ligaments, and fascia that act as a sling to support the bladder, reproductive organs, and rectum^1)^. Superficial PFMs include the bulbospongiosus, ischiocavernosus, transverse perineal muscles, urethral sphincter, and anal sphincter. Deep PFMs that line the inner walls of the pelvis include the levator ani and coccygeus which, together with the endopelvic fascia, comprise the pelvic diaphragm. The levator ani muscle is not a single but has two functional components that vary in thickness, origin, and function^14)^. The iliococcygeus has a mainly supportive function, whereas the puborectalis has a sphincteric function.

In humans, androgen responsiveness of the levator ani muscle is evolutionarily conserved and is significantly reduced compared with other muscles in men receiving androgen deprivation therapy^15)^. PFM volume was measured using magnetic resonance imaging (MRI). However, details of the PFM measurement method are not yet established. The weight of the levator ani muscle can be measured in mice. On the other hand, it is impossible to measure the weight of PFMs in living human. As an objective indicator of PFMs instead of weight, Sadahira et al. reported that preoperative thickness of the anorectal muscles helps improve post-RARP urinary incontinence^16)^. Li et al. reported that the distance of the inner part of the levator ani muscle was considered as a factor to predict urinary continence after RARP by using the method of inserting preoperative MRI into an artificial intelligence^17)^. In addition, the measurement of PFM thickness using ultrasound and the measurement of the LH distance, thickness, and area using MRI have been reported as simple objective indicators of PFM^18, 19)^. The LH may be assessed to indirectly evaluate the PFMs. Narita et al. measured the LH area in healthy volunteers using CT and revealed that it was significantly larger in the standing position^11)^. LH is 10% larger when measured in the axial plane of the body than when measured parallel to the direction of the puborectalis^20)^. Whether PFM evaluation based on distance or area reflects PFM function remains unclear. It may be important to assess PFM by volume.

A working group has recommended a method for evaluating the pelvic floor using MRI^21)^. Thus, an ultrasound gel is injected into the rectum, abdominal pressure is applied, and dynamic MRI is performed to accurately diagnose POP in women. This test is somewhat invasive to perform prophylactically. Rodrigues et al. reported on the measurement of levator ani muscle volume using MRI in women^22)^. They used a 3D slicer software package to measure the levator ani muscle between the pubococcygeal and hiatus lines. They have also developed an estimation formula for levator ani muscle volume based on the WLH, M line, and H line values. They reported to have spent 2 hours on volume measurement for a patient, which is excessively time consuming for preventive imaging. Male PFM volume was measured using CT, a more convenient examination method in our study. The time required for a patient was about 10 minutes. The reliability of the measurement of PFM volume was confirmed. CT is easier to perform than MRI and therefore more versatile. However, it may be necessary to measure PFM volumes in the same patient using both MRI and CT to verify validity. Muro et al. showed anatomical evidence that hip motion with the obturator internus contributes to voiding and urinary function^23)^. The bony and muscular pelvis is highly interconnected to the hip and gluteal musculature, together supporting the internal organs and core muscles^1)^. In addition to PFMs, the obturator internus is another muscle that should be evaluated further in the future.

We also evaluated the myosteatosis of PFMs by the CT attenuation. In recent years, image evaluation using CT and MRI has been used to evaluate sarcopenia^24)^. The cross-sectional area of the psoas major muscles bilaterally at L3 or L4 and the cross- sectional area of all muscles at the L3 level are often used to evaluate sarcopenia. Increased fatty infiltration of muscle reduces the radiodensity of muscle on CT images. Fatty infiltration of PFM may be important in the evaluation of urinary and sexual function. The relationship between the decrease in CT value and PFM volume is interesting. The area of the LH, H line, and M line differed between younger and older patients. Potential POP in men may also be associated with urinary and sexual function. The B line is not an appropriate indicator because it is affected by benign prostatic hyperplasia in men. The PCL angle was greatest in younger patients. The bony anatomy of the pelvic girdle consists of three bones (two innominate bones and the sacrum) and three joints (the sacroiliac joints and the pubic symphysis). Yoon et al. reported that the sacrum tended to curve and the coccyx tended to straighten with age^25)^. They also reported that the coccyx was straighter in women than in men. The PCL angle is related to the shape of the coccyx. Although the coccyx has an innate type, as the PFM descends with age, the sacrum and coccyx may be pulled forward and downward, resulting in a smaller PCL angle. The relationship between the PCL angle and PFMs needs further validation in the future.

A limitation of this study is that not all PFMs were measured. Areas related to sexual and urinary functions may be part of the PFM we measured. It is difficult to capture the contractions of these small muscles using CT or MRI dynamically. To clarify this, it may be useful to evaluate the contractions of individual muscles during urination using ultrasound. This is an issue that needs to be considered in the future. This study focused on the methodology for measuring PFM volume, and evaluated its reliability using the calculated minimum sample size. We are currently conducting a large-scale study using this methodology.

In conclusion, the high reliability of male PFM volumes using 3D-CT was confirmed. This study can provide objective indicators for the PFMs evaluation. In the future, evidence including myosteatosis in male PFMs will be accumulated.

## Funding

This study was supported by JSPS KAKENHI (Grant Number: JP23K10436).

## Author contributions

FS contributes to the conception of study design, AA instructed how to use the workstation, MD and YT (YTanaka) performed the data acquisition, FS and TK analyzed the data, HI, YT (YTamura) and SH were supervised the methodology.

## Conflicts of interest statement

The authors have no conflict of interests to declare. Yoshifumi Tamura, one of the Editorial Board members of JMJ was not involved in the peer review or decision-making process for this paper.
